# Society Proceedings

**Published:** 1899-01

**Authors:** 


### SOCIETY PROCEEDINGS.

### THE MONTREAL VETERINARY MEDICAL ASSOCIATION.

THE December meeting of the Association was held in the library of the college, the Vice-President, Dr. Baker, occupying the chair.

After the minutes of the last meeting had been read, Mr. Hammond reported the following case: The subject, a red cocker-spaniel, was brought to Mr. Hammond, suffering great pain, and with a history of having lost weight rapidly. Examination revealed the presence of a movable tumor, which at times rested between the jaws, and at others lay upon the trachea, causing intense dyspnœa on the slightest exertion. Mr. Hammond tried painting the part with the tincture of iodine, persisting in this treatment for a couple of days, but as the animal became worse an operation was suggested, to which the owner readily consented. The field of operation being denuded of hair, was washed with soap and water and then with a 1 : 100 formalin solution. An anæsthetic having been given, an incision two inches long was made over the swelling, entering a large, suppurating mass, and in cutting further two more growths were encountered, which bled profusely, demanding the use of forceps for its control. The third portion of the tumor wound around the carotid artery. After removing the growths the wound was washed with a 1 : 20 carbolic acid solution, then closed with catgut sutures and dressed with lint saturated with collodion in which iodoform had been dissolved, the whole being covered with a bandage. Five days after the operation the stitches were removed and the animal sent home in good health, free from pain of any kind. Examination of the tumor showed it to be of a fibrous nature, with a few cysts in which were found pus and blood-clots.

After some discussion in the case, Mr. Henderson exhibited the lower jaw of a five-year-old ewe which had been sent to him, saying that the case was one he thought very rarely met with in an ordinary everyday practice, and one which should merit discussion, although no clever diagnosis or even remarkable cure is connected with it. The subject, a five-year-old ewe, had been in poor health for some time and had undergone treatment which was in no way beneficial. The owner, being of a sympathetic nature, decided to destroy the animal and examine the lower jaw, which was very much swollen. On examination he found the cavity, shown in the specimen, filled with semi-masticated grass. He decided to forward the specimen from British Columbia for Mr. Henderson's inspection, adding the above history; also asking for a full report as to the cause, of any indicated treatment; also if due to any contagious disease which was apt to affect the remainder of his flock. On examining the specimen, enlargement involving both inferior maxilla were found, but more pronounced on the right maxilla. There was also a large cavity extending from before, inward and backward, the teeth being very much displaced, the same being totally reversed. The last molar tooth in the right maxilla has protruded through on the inferior side, and shows a worn appearance. There is also a deterioration from the normal shape on the inferior-internal side of the right maxilla, the teeth being also displaced on the opposite maxilla, and show very uneven wear and a tendency to become like the other side. In concluding, Mr. Henderson said that he presumed this case had been one of difficult dentition or caused by a blow of some kind received under the jaw.

Mr. Groves then presented his essay on "Horseshoeing," beginning by briefly describing it from its earliest days, in the fifth or sixth century B. C., where iron was first used for the purpose of making shoes, down to modern times, when there are a thousand and one patent shoes on the market. The anatomy and physiology of the foot were then minutely and accurately described, after which the matter of shoeing the foot in its normal and diseased conditions were carefully related. The many and varied methods of correcting abnormal gaits in the trotting horse were described, the latter part of the essay proving of a special interest to the number present. A discussion followed, in which Messers. Paquin, Stanbridge, and Gellably took an active part. Mr. Groves proved himself able to reply to all questions, and displayed a perfect mastery of the subject. After a few remarks from the chairman on shoeing in general, the meeting adjourned.

JAMES McGREGOR,
Secretary-Treasurer.

### CALIFORNIA STATE VETERINARY MEDICAL ASSOCIATION.

THE regular meeting was held at the Palace Hotel, San Francisco, December 14, 1898. The meeting was called to order by President Dr. R. A. Archibald. Upon roll-call the following-named gentlemen responded: Dr. H. A. Spencer, Dr. H. F. Spencer, Dr. R. A. Archibald, Dr. F. E. Pierce, Dr. Graham, and Dr. C. L. Megowan.

The minutes of the previous meeting, as also reports of the Secretary and Treasurer, were read and approved.

The next order of business was election and installation of officers, which resulted as follows: President, Dr. R. E. Pierce; Vice-President, Dr. G. F. Faulkner; Secretary, Dr. C. L. Megowan; Treasurer, Dr. J. Graham. Board of Examiners: Drs. Archibald, McCollum, Graham, and Pierce. After a few very appropriate remarks from Dr. Archibald, the chair was filled by Dr. F. E. Pierce, other newly elected officers taking their respective offices.

Under the heading of good of the organization, Dr. H. A. Spencer addressed the members present very briefly and thoughtfully, referring to years gone by and the rapid progress veterinary science had made since he became a member of the association, and the benefit derived therefrom. President Dr. Pierce stated that inasmuch as it was nearing lunch time he would entertain a motion to adjourn until 8 P.M., which was adopted; whereupon Dr. Pierce tendered an invitation to the members to partake of the most luscious foods and drink. After a most bountiful repast the members retired to the meeting room for the discussion of many important matters and to listen to the humorous stories tendered by Dr. Spencer, Jr. President Dr. Pierce called the meeting to order, and reading of papers and the discussions were announced as in order.

Dr. R. A. Archibald read a most valuable paper on the subject of "Texas Fever," exhibiting the different varieties of ticks, and gave a most interesting account of their life-history. Discussions followed, in which all members took part.

After listening to numerous reports of cases, the subject of legislation was brought up, and the matter was thoroughly discussed both by members of the Association and other prominent men interested in livestock.

On motion of Dr. C. L. Megowan, Dr. R. A. Archibald was tendered a vote of thanks for his most excellently prepared paper on the subject of Texas fever.

There being no further business, the meeting adjourned to meet in San Francisco, March 8, 1899.

C. L. MEGOWAN, V.S.,
Secretary.

### CHICAGO VETERINARY SOCIETY.

THE regular monthly meeting was called to order December 8th, at 8.30 P.M., President Robertson in the chair. The roll-call showed sixteen members present, Dr. Butterfield visiting by invitation of Dr. B. A. Pierce.

The minutes of the previous meeting were read and approved. President Robertson passed over the usual remarks in order to expedite the business of the evening. The reports of the Secretary and Treasurer were dispensed with, and a communication from Dr. W. J. Martin, President of the Illinois State Veterinary Medical Association, calling attention to the great need of veterinary legislation in this State, was read and referred to the Committee on Legislation.

The application of Dr. W. E. Howe, a graduate of Ontario Veterinary College, 1896, and New York State Veterinary College, 1897, was favorably reported back from the Board of Censors, and his admittance to membership was voted on and carried.

A communication from a committee representing the Iowa and Nebraska State Veterinary Medical Associations, asking this Society to consider the advisability of organizing and becoming a part of the Trans-Misssissippi Veterinary Medical Association, which was laid over from the November meeting for consideration, was re-read and discussed, which resulted in the Secretary being instructed to inform the committee that this Society as a body would take no action; this decision of the Society not to be construed as an indication of unkind feeling toward the matter, as individually the members recognized the vast good derived from veterinary societies, and looked very favorably upon the proposed organization.

Dr. H. W. Hawley's paper met with enthusiastic praise for its originality. A lively discussion followed, in which the following took part: Drs. Merillat, Hughes, Ryan, Quitman, Robertson, Walker, Campbell, and Pierce. Dr. A. M. Casper was to have presented a paper on "Temperature," but he failed to appear.

Responding to a call of the President, Dr. L. A. Merillat described a "New Method of Operating for Abscess of the Guttural Pouch," which was of much interest.

Dr. Allen reported a "Case of Hoof Sloughing Following High Neurectomy." Dr. Hughes mentioned two cases of sloughing of hoofs in mules following the same operation, and gave as his opinion that every time you neurectomize a mule you can expect a sloughing.

*Dr. Hawley's Paper on Vices and Their Relation to Soundness.*

I shall define a vice in a horse as a fault or habit which injures the selling price or usefulness of the animal for the purpose required, and which cannot be remedied. I shall name cribbing, weaving, balking, kicking, shying, halter-pulling, switching, side-reining, lugging, running away, and nervousness as vices. Vices are natural, hereditary, or acquired.

*Cribbing* is a habit, the result of idleness and too long intervals between meals. The horse amuses himself, or is hungry and bites at the manger, and gradually works himself into this habit. A cribber should in every case be rejected, as it is a very disagreeable habit, leading to indigestion and kindred diseases. The animal is also liable to disfigure or destroy anything with which its teeth come in contact. Wedges driven between the teeth will produce sufficient soreness in a few hours to prevent cribbing temporarily. All suspicious cases should be examined for this trick.

*Weaving* is also a habit acquired from idleness. The rattle of a chain halter may assist in the formation of the vice. Circumstances might determine the acceptance or rejection of a weaver. A horse does not weave in harness, at least I never seen one. There is slight danger of it injuring the health of the animal, especially if he is in constant service. After explaining the nature of a weaver to the buyer he should be advised to use his own judgment. The animal might be valuable as a prize-winner, sire, or brood-mare.

*Balking.* A man who has patience enough to use a balky horse deserves to be pensioned. I have never known of a balky horse being cured of the habit. He is liable to stop at any moment, and if stopped may refuse to move, and should by all means be rejected. We have what may be termed green balkers. The horse may be clever in double harness, but when first put in shafts will manifest all the symptoms of a balker, but soon becomes clever with a lesson or two. Unless sold to be thoroughly broken to single harness, such a horse should not be permanently rejected. A good test for a balky horse is to start, stop, back up, and then start again. If he does these perfectly he is probably all right.

*Kicking.* A kicking horse is certainly not a very desirable animal, either in the stall or in harness. A horse when first put in single harness may kick a few times, but soon becomes gentle with a little handling. Mares sometimes kick in double harness, but work clever single. A kicking harness horse might be useful as a saddler if sold for that purpose only.

*Shying.* Nearly all country horses shy when first brought to the city, but some are short-sighted and always shy. The latter should be rejected. But unless a horse is sold to be thoroughly city broken, he should not be rejected for shying unless it can be determined it is chronic.

*Halter-pulling.* A confirmed halter-puller might be useful as a coach horse if kept in a box-stall. In such a case the buyer may be allowed to use his own judgment.

*Switching.* This is rarely seen in the horse, and is the result of disease of the ovaries in mares. They should be rejected.

*Side reining* is one of the most disagreeable vices a horse can have. If it is caused by a sore mouth, and it can be shown that it is only temporary, the horse might be seen again after the mouth heals.

*Lugging* is also a disagreeable habit, but cannot by any means always be called a vice. A horse may lug on one kind of a bit and drive beautifully with another. It is not an unsoundness, as a horse will not lug when properly hitched.

*Running away.* There is a difference of opinion as to what constitutes a runaway horse. Any prompt, free driver will run away if unrestrained. Some first-rate family horses will run away under certain circumstances. Some will say that a horse that has once run away is never safe again. Such is not the case. A horse that will suddenly make a dash for liberty, and which cannot be controlled with the ordinary driving-bit, is in my opinion a runaway horse. In examining horses for soundness it is customary in this country to run them for their wind in harness. If they stand such a test with an ordinary bit they should be passed.

*Nervousness* is hereditary, but a high-bred animal becomes nervous with abuse. Some may be useful for some purposes and totally unfit for others. What one person would call a nervous horse would just suit another. I know a veterinarian who could not be induced to ride behind a snappy, high-class horse. I know another who would not give a dollar for a horse unless he looked wilder than a hawk. The practical examiner should consider the person and purpose for which the horse is bought.

Leaving the subject of vices for discussion, I will conclude with a few remarks on the practical part of examining horses for soundness. I take it that most of the members present are trying to enlarge their bank account. The price of examining horses is from $2.50 to $7.50. It takes very little time and labor to look over a horse, and a hundred or so each year examined will go quite a distance in the payment of expenses.

It has been argued that the dealer or seller should receive no consideration whatever. I have observed that about 75 per cent. of the horses sold in Chicago subject to veterinary examination go before one man. I said to myself, "there is some reason for this;" so when I saw him look at some horses I tried to discover the secret of his success, and I found that it was his fair treatment and consideration of the seller as well as the buyer. In fact, he has the universal respect of every reputable dealer in the city. They know he will not reject their horses, provided they are useful for the purpose required. Another thing I noticed was that he apparently has no whims or prejudices, and is not married to any particular breed or color. The result of this is that every dealer in the city is ready to sing his praises at every opportunity, which goes a long ways toward keeping up his reputation.

I know another veterinarian who is universally disliked by the dealers, and they will miss the sale of their horses rather than see them go before him for examination. He starts out in his examination with the idea of finding something on which to reject a horse, and usually accomplishes his aim.

Another veterinarian is afraid almost to pass a horse for fear something will happen for which he will receive the blame. I once received a horse from the country which was a fine specimen of his class. His conformation was superb, his action faultless, he was a coach-horse in every sense of the word, and such a horse as one would strive for months to find. He had a small, insignificant splint, slight wire-mark on the pastern, his mouth had been forced probably three months to a five-year-old, and there was a small scar on the cornea of the left eye. Many offers were received for the horse, and finally he was disposed of for six hundred dollars to a man on the North Side. This man had been looking for two years for just such a horse. He bought him subject to veterinary examination. The doctor overlooked the splint, spot in the eye, and wire-mark, but called him a four-year-old. The gentleman, hating to part with such an animal, called a second doctor, who passed him on age, speck in the eye, and splint, but could not stand the wire-mark. The third doctor was called, who accepted the wire-mark and age, but noticed the speck in the eye, and the splint would surely produce lameness inside of six weeks. The horse was returned to me, and I was branded as a swindler. I told the gentleman to take the horse and use him three months if he liked. If at any time he felt like paying for him to do so, otherwise to return him to me at the end of the three months, and to pay whatever he liked for the use of him. The horse has been paid for, and is one of the finest on the North Side to-day. No veterinary surgeon will ever examine a horse for that man again. So good a buyer as Mr. John Dupee has dispensed with veterinary examination entirely, and now examines his own horses. His reason is that they kept him from buying any good horses.

There is a class of dealers who work nothing but a skin-game; but their horses seldom, if ever, go before a veterinarian. They know what will happen. There is another class of dealers who handle nothing but the very best, and every horse is sold on a guarantee, and they will make their guarantee good. For a veterinarian to reject one of their horses on a mere technicality or personal whim is to drive a nail in his own professional coffin. The chances are the horse will be sold in spite of his opinion, and should it prove satisfactory the buyer loses confidence in the doctor.

I know a doctor who has noticed all his life that horses with three white legs are of little practical value. The specifications for cavalry horses say a gelding of uniform and hardy color; when, as a matter of fact, a mouse-colored horse with a black stripe down his back, a buckskin, or a roan, is no better or tougher than the soft bay, ginger-bred sorrel, or the despised horse with three white legs.

Veterinarians are criticised by dealers more than by any other class of people, and one who does not go about a horse in a horseman-like manner is put down as a professional fool, and is so advertised. A short time ago a doctor holding a very prominent position with the United States Government was called to the stockyards to examine a draught-horse. The horse had a pronounced curb, which could be plainly seen quite a distance. There was a crowd of horsemen about, every one of whom saw the curb. The horse was not expected to pass the doctor, and it was supposed he would reject him at first sight. He spent nearly half an hour examining every point about the horse, and finally by accident saw the curb and rejected him. A smile passed over the faces of the horsemen, and the rest of the veterinary profession no doubt had to suffer for this man's shortcomings. I could mention many other absurdities which I have observed, but these will suffice.

The following is my method of examining a horse for soundness: First of all, I never examine a horse in harness, as it covers defects, and the horse may have been warmed out of lameness. I watch him as he is being backed from the stall, to see that he is not vicious, a halter-puller, or crampy. Standing at a reasonable distance, eight or ten feet, or walking around the horse, a general view of the outlines can be had. In this way any prominent defect is discovered, and it is not necessary to go any further. Not discovering anything, the horse is led to the door. Just before passing out of the door I examine the eyes and mouth. The light at this point is just right to reveal any defects of the eyes. The horse is then walked and trotted at the halter. I always stand directly in front when the horse is coming toward me, watching the front limbs only, likewise the hind ones as he goes away. In passing his knee-action and hock-action are observed. His gaits being perfect, I then examine him in a methodical manner, commencing at the nose and going over each section. This is repeated on the opposite side. He is then turned short and backed for string-halt, after which he is put in harness and run for his wind.

Dr. Campbell: Dr. Hawley mentioned the case of a veterinarian who was very much liked. What would he do in the case of the six-hundred dollar horse that had the splint?

Dr. Hawley: He would pass the horse. I mentioned that the horse had but a small, insignificant splint.

Dr. Merrillat: I would like to have Dr. Hawley again describe his remedy for a halter-puller.

Dr. Hawley: It is very simple. A rope is passed around the body of the animal, over the back and between the front legs, and then through the halter-ring, to be fastened to the manger.

Dr. Merrillat: This paper is one of the best that I have ever listened to, as it is full of practical suggestions that are original deductions from personal experience, and I hope it will be published. There is one point about side-pulling on which I have my own ideas. The doctor mentioned that side-pulling is due more to a sore mouth than to anything else, while I find it to be quite different. In many side-pullers we do not find anything wrong. I think it is due to the cleverness of the horse. He is naturally ambitious, and knowing that when he turns his head a little to one side he can take advantage of the driver, he wants to go ahead as fast as the driver is willing to allow him. The remedy is simply. An apparatus consisting of a leather washer with tacks driven through it generally cures the horse. There is also another side-puller that ought to be called side-goer. He does not pull much, but insists on going to one side of the street. That horse is incurable, as far as my experience goes. No matter what you do for him, he continues to go to one side; therefore, I consider side-goers and side-pullers as quite different, and believe the former to be incurable.

Dr. Walker: I am quite interested in Dr. Hawley's paper, and acknowledge that it is one of the best that I have ever heard. He states that he never examines a horse in harness. What would he do in a case where the owner refuses to have the harness removed? In examining a one-thousand dollar horse, for instance, do you think by taking the horse out for fifteen minutes you could determine that the animal is not crampy? Would it not be better to have him in your possession for at least twenty-four hours?

Dr. Hawley: I referred to the absurdities indulged in by some veterinarians in making an examination for soundness. Very often they make a laughing-stock of themselves. Certainly the value of the animal has some bearing upon the length of time spent in making an examination. In examining a one-thousand dollar horse I would naturally be more particular than with a fifty-dollar horse; and in a high-priced horse it is preferable to have him in my possession for at least twenty-four hours. As far as examining them in harness is concerned, there is no dealer who wants to do what is fair that ought to object to its being taken off. No horse should be examined with the harness on, as it covers many defects.

Dr. Walker: A veterinarian has to be very careful with horsedealers. One of my clients went to one of the supposed-to-be reputable horsedealers to look at a horse. The dealer pronounced the animal perfectly sound. My client offered him twenty dollars down, the balance to be paid upon my examining him and pronouncing the animal sound. To this the dealer objected, saying that I had a dispute with him over an animal before. This was not true. Another veterinarian was called, who pronounced the animal unsound.

Dr. Quitman: Regarding the six-hundred dollar horse, you mention that he had a scar on the cornea. Can you be positive whether it was a scar or an opacity?

Dr. Hawley: Yes.

Dr. Quitman: I examined a horse some time ago—an ordinary working horse. He was sound in every particular, except he had a very small opacity in the cornea. I examined him carefully to see if any signs of periodic ophthalmia were present. I passed the horse, and regret it ever since. That horse had periodic ophthalmia.

Dr. Hawley: Why did you pass the horse?

Dr. Quitman: To do justice to the dealer as well as to the buyer. We often find horses with such opacities, or rather specks, which are all right. Possibly this horse was prone to periodic ophthalmia.

Dr. Ryan: In regard to this speck in the eye, I think any scar or speck causes a defect of vision. If it is very close to the sclerotic it should not cut much figure; but if there is any speck within the cornea I would not hesitate to pronounce him unsound.

Dr. Hughes: I think we should all join Dr. Merrillat in congratulating Dr. Hawley on his paper. It deserves every consideration, and I hope to see it published. I would like to hear an expression of opinion in regard to the nature of cribbing; I would like to know whether it is possible for such an animal to actually swallow air and inflate himself. With regard to weaving: what is a weaver, anyhow? What is the cause of it? Is it a nervous disease? If it is, what authority is there for saying so? Physicians often ask these qustions, and it is a slur on us not to be able to answer these questions satisfactorily.

Dr. Campbell: Dr. Merrillat will read on weaving and cribbing.

Dr. Hughes: If such is the case I withdraw my questions.

Dr. Robertson: In regard to the scar in the eye, there is another thing I would like to know about. There are some operators who operate on periodic ophthalmia. At the time of operation there was a distinct scar on the eye. The operator assured me that after he operated on the eye there would be positively no return of the ophthalmia. Even if the eye was clear from opacity, and only the scar from the operation remained, I am dubious whether it would be justifiable to pass such a horse. I had a horse with a speck in the eye, and he would at times jump as if surprised by something, being due to interference with his vision; and I think that a veterinarian cannot be too careful in such cases; and the attention of the buyer should be called to such specks or scars whenever there are any.

Dr. Quitman: How was this operation you refer to performed, and how would it prevent further ophthalmia?

Dr. Hughes: In five cases that I operated upon three of them had subsequent attacks. One of them had no subsequent attack, while the fifth one I have not been able to keep track of. The operation is very simple: Make an incision at the cornea and sclerotic junction and liberate the contents of the anterior chamber. The eye collapses when the humor is liberated, but fills up again in four or five days.

Dr. Hawley: In regard to Dr. Ryan's statement as to his pronouncing a horse with a speck in the eye unsound, I do not want it understood that I was in any way criticising or defending the opinion of the veterinarians. I just wanted to show the effect that these opinions have on the buyer. One rejected the horse on account of the eye, while the other passed him.

Dr. Robertson: Is there any member who has anything new in the way of operations to report?

Dr. Merrillat: About a year ago we had presented at the clinics a case of chronic nasal discharge, which was diagnosed as pus accumulation in the guttural pouches. The animal was cast and the pus removed by operating through the fauces. Since that time I have made a number of experiments along this line. We have shown that by cutting the soft palate in the median line throughout its whole extent operations in the pharynx are comparatively simple. The hand containing a curved bistoury is passed through the fauces, and the soft palate is cut from behind forward, from the base of the epiglottis to the palatine bone. The fingers can then be inserted into the Eustachian tubes with ease, and an examination of the guttural pouches easily made by palpation. If they are found to contain pus the roof of the pharynx is cut in the median line. This admits the fingers between the guttural pouches. The abscess is then broken into with the fingers. The first operation was performed on an animal in a recumbent position, but since that time a number have been operated upon in the standing position. The soft palate never reunites, but the animal seems to suffer no inconvenience therefrom. This new procedure of cutting the soft palate certainly opens a new field for diagnosis if not for a number of surgical operations. The operation can be performed in a standing posture by the aid of a good, substantial speculum.

### VETERINARY MEDICAL ASSOCIATION OF NEW YORK COUNTY.

THE regular annual meeting of the Association was called to order Wednesday, December 7th, in room 37, New York Academy of Medicine, at 8.45 P.M., by President Robertson. On roll-call the following members responded: Drs. Ackerman, Bell, J. S. Cattanach, Dickson, Delaney, Ellis, Farley, Gill, Grenside, Hanson, Lellman, O'Shea, and Robertson. The minutes of the previous meeting were then read, approved, and ordered placed on file. The Board of Censors then recommended for membership in the Association the names of Charles E. Clayton, D.V.S., graduate of the American Veterinary College, whose application had come before them in regular form, with Robert W. Ellis and James L. Robertson as vouchers, and George J. Goubeaud, D.V.S., graduate of the American Veterinary College, whose application was also in regular form, with E. B. Ackerman and R. R. Bell as vouchers. An application for honorary membership in the Association, approved by the Board of Censors, was also presented, as follows:

ACADEMY OF MEDICINE, NEW YORK CITY, December 7, 1898.

According to Article X. of the Constitution and By-laws of the Veterinary Medical Association of New York County, we hereby propose for honorary membership Hon. Timothy P. Sullivan, of New York County, Borough of Manhattan, the gentleman who aided this Association in the passage of the bill exempting veterinary surgeons from jury duty. He had the bill passed in the Assembly this year, 1898. Signed, Arthur O'Shea, H. D. Gill, Roscoe R. Bell, J. L. Robertson.

Drs. Clayton and Goubeaud were declared members of this Association and Hon. Timothy P. Sullivan an honorary member of the same.

Dr. Lellman then read a very scientific paper on "Hydrocephalus in the Horse," with microscopical demonstrations, the specimens being exceptionally fine. After the discussion, which followed, a vote of thanks was tendered to Dr. Lellman from the Association, as well as one personally expressed by the President.

Dr. Goubeaud, of the Borough of Brooklyn, then read a paper entitled "A New Method of Employing Charcoal in the Treatment of Acute Indigestion in Horses." This paper opened up a field for animated discussion, which was pretty generally indulged in by the members.

A hearty vote of thanks was tendered to Dr. Goubeaud. The Judiciary Committee having nothing to report, the Committee on Resolutions on the death of Dr. Machan was called upon the President, and offered the following report:

*Whereas*, It has pleased the Almighty to remove from our midst Dr. William Machan; and

*Whereas*, Our relation with him in the veterinary profession makes it fitting that the members of this Society record their appreciation of him; therefore, be it

*Resolved*, That the very sad and sudden removal of such a man leaves a vacancy that will be deeply realized by the members of the profession;

*Resolved*, That this be spread in full in the minute-book, a copy be sent to the relatives, and be published in the veterinary journals.

Signed, H. D. Hanson, D.V.S., Chairman; H. D. Gill, V.S., J. L. Robertson, M.D., V.S.

Moved and sconded that the same be accepted and placed on file; carried.

Ways and Means Committee, Dr. Bell, Chairman, promised to continue their very interesting programme, samples of which the Association have been enjoying for several meetings past.

The usual routine of business being concluded, and this being the annual meeting, election of officers for the ensuing year, 1899, was next in order. Dr. H. D. Gill, after a very few neat and fitting remarks, nominated for President Dr. J. L. Robertson, the nominations being immediately closed. The By-Laws were suspended and Dr. Robertson was elected by acclamation. The same course was pursued with the rest of the nominees, which resulted in the election for Vice-President of Dr. H. D. Gill; for Secretary, Dr. Robert W. Ellis, and for Treasurer Dr. H. D. Hanson.

Dr. Hanson, who had served in the capacity of Treasurer during the year just ended, then rendered his report.

Moved and seconded that the report be accepted.

Moved and seconded that the meeting adjourn.

ROBERT W. ELLIS, D.V.S.,
Secretary.

### AMERICAN VETERINARY MEDICAL ASSOCIATION.

*List of Resident State Secretaries:*

Alabama—R. H. Drummond, Birmingham.
Arizona—J. C. Norton, Phœnix.
California—Fred C. Pierce, 1724 Webster St., Oakland.
Connecticut—R. P. Lyman, 328 Asylum St., Hartford.
Delaware—H. P. Eves, 507 W. Ninth St., Wilmington.
District of Columbia—A. M. Farrington, Dept. of Agr., Washington.
Georgia—George B. Blackman, Rome.
Illinois—E. M. Nighbert, Mt. Sterling.
Indiana—J. R. Mitchell, Evansville.
Iowa—T. A. Bown, Chariton.
Kansas—W. N. D. Bird, Arkansas City.
Kentucky—J. W. Jameson, Paris.
Louisiana—W. H. Dalrymple, Baton Rouge.
Maine—G. N. Bailey, Deering.
Maryland—Wm. Dougherty, 1035 Cathedral St., Baltimore.
Massachusetts—E. H. Holden, Springfield.
Michigan—S. Brenton, 83 Fifth St., Detroit.
Minnesota—C. E. Cotton, Minneapolis.
Mississippi—J. C. Robert, Agricultural College.
Missouri—Charles Ellis, 3230 Locust St., St. Louis.
Montana—M. E. Knowles, Butte.
Nebraska—V. Schaefer, Tekamah.
New Hampshire—Lemuel Pope, Jr., Portsmouth.
New Jersey—J. P. Lowe, Bloomfield Ave., Passaic.
New York—Wm. Henry Kelly, 195 Western Ave., Albany.
North Carolina—A. S. Wheeler, Biltmore.
North Dakota—T. D. Hinebaugh, Fargo.
Ohio—T. Bent Cotton, Mt. Vernon.
Pennsylvania—W. H. Ridge, Trevose.
South Carolina—Benj. McInnes, Charleston.
South Dakota—M. J. Treacy, Fort Meade.
Tennessee—Joseph Plaskett, 529 Broad St., Nashville.
Texas—M. Francis, College Station.
Virginia—E. P. Niles, Blacksburg.
Washington—S. B. Nelson, Pullman.
West Virginia—L. N. Reefer, 1406 Chapline St., Wheeling.
Canada—W. J. Hinman, Winnipeg Manitoba.

### ONTARIO VETERINARY ASSOCIATION.

THE annual meeting was held in the Veterinary College, Toronto, Canada, on Friday, December 23, 1898.

The President, Professor S. Sisson, called the meeting to order at 11.10 A.M., and after a short address the minutes of the previous meeting were d and confirmed.

Papers and communications received by the Secretary since the last meeting were then read and discussed.

The auditors reported the finances of the Association to be in a healthy condition, there being funds on hand.

The following new members were then duly elected: Mr. H. S. Perley, V.S., Ottawa; Mr. J. H. George, V.S., Ingersol; Mr. P. G. Button, V.S., Stouffville; Mr. F. Bryant, V.S., Sunderland; Mr. R. E. Willis, V.S., Woodbridge.

A resolution of sincere sympathy with Mrs. C. L. Smith, in the loss of her husband, Mr. C. L. Smith, V.S., an esteemed member of our Association, who has just been called away in the midst of his usefulness, was passed, and the meeting adjourned for lunch.

The meeting was opened again at 1.30 P.M.

The reading and discussion of papers was the first order of business.

Mr. W. J. Wilson read an interesting paper on "Thrombus," which was well discussed.

Mr. O. Graham read a good paper on "Veterinary Science in Relation to Public Health," and in the course of the discussion that followed Professor A. Smith read an extract from Dr. McEachran's interview with Ostertag, the leading authority in Europe on milk- and meat-inspection.

Professor Sisson, Demonstrator of Anatomy, read an excellent paper on the "Synovial Membranes of the Tendons." These he demonstrated on the blackboard, and also exhibited some beautiful specimens from the Museum of the Ontario Veterinary College that were prepared by Mr. J. F. J. Black, a student, who passed two years ago.

Mr. W. Lawson brought up the subject of better legal protection for the profession in Ontario, and after a long and animated discussion it was ultimately resolved that the following clause should be submitted to the Provincial Parliament to be inserted in the act of incorporation of the Ontario Veterinary Association: "It shall not be lawful for any person not registered to practise veterinary medicine or surgery or to perform any surgical operation on animals, for hire, gain, or hope of reward; and if any person not registered pursuant to this act, for hire, gain, or hope of reward practises or professes to practise veterinary medicine or surgery, or advertise to give advice in veterinary medicine or surgery, he shall, upon a summary conviction before any justice of the peace, for each and every such offence, pay a penalty not exceeding twenty-five dollars nor less than five dollars." The Secretary was instructed to get a number of copies printed and distribute them to graduates in Ontario, with a request to each to see the member of the Provincial Parliament for his locality and induce him to favor the proposed legislation.

The sum of twenty-five dollars was appropriated for a medal to be competed for by the students of the Ontario Veterinary College at the examinations in the spring.

The following are the officers of the Association for the ensuing year: Professor S. Sisson, President; Mr. W. J. Wilson, First Vice-President; Mr. J. Blackall, Second Vice-President; Mr. C. H. Sweetapple, Secretary and Treasurer. Directors: Messrs. W. Steele, J. Wagner, G. Hilton, O. Graham, W. Lawson, G. Coulter, F. Bryant, and J. H. George. Auditors: Messrs. C. Elliott and J. D. O'Neil. Delegates to the Industrial Fair Association: Professor A. Smith and the President. Delegates to the Western Fair Association: Messrs. J. H. Wilson and J. D. O'Neil.

C. H. SWEETAPPLE,
Secretary-Treasurer.

